# *Anaplasma phagocytophilum* in Ticks Blood-Feeding on Migratory Birds in Sweden

**DOI:** 10.3390/microorganisms12040735

**Published:** 2024-04-03

**Authors:** Peter Wilhelmsson, Malin Lager, Thomas G. T. Jaenson, Jonas Waldenström, Björn Olsen, Per-Eric Lindgren

**Affiliations:** 1 Division of Inflammation and Infection, Department of Biomedical and Clinical Sciences, Linköping University, SE-581 83 Linköping, Sweden; per-eric.lindgren@liu.se; 2 National Reference Laboratory for Borrelia and Other Tick-Borne Bacteria, Division of Clinical Microbiology, Laboratory Medicine, Region Jönköping County, SE-551 85 Jönköping, Sweden; malin.lager@rjl.se; 3 Department of Organismal Biology, Evolutionary Biology Centre, Uppsala University, SE-752 36 Uppsala, Sweden; thomas.jaenson@ebc.uu.se; 4 Centre for Ecology and Evolution in Microbial Model Systems, Linnaeus University, SE-392 31 Kalmar, Sweden; jonas.waldenstrom@lnu.se; 5 Zoonosis Science Center, Department of Medical Sciences, Uppsala University, SE-751 23 Uppsala, Sweden; bjorn.olsen@medsci.uu.se

**Keywords:** *Anaplasma phagocytophilum*, migratory birds, human granulocytic anaplasmosis, zoonosis, tick-borne fever, Sweden

## Abstract

Migratory birds play a dual role as potential reservoirs of tick-borne pathogens, and potential dispersers of pathogen-containing ticks during their migratory journeys. *Ixodes ricinus*, a prevalent tick species in Northern and Western Europe, serves as a primary vector for *Anaplasma phagocytophilum*—a bacterium with implications for human and animal health. There is limited information available regarding *A. phagocytophilum* in birds. Our investigation focused on *A. phagocytophilum* prevalence in ticks collected from migratory birds in southeastern Sweden. The identification of ticks involved both molecular analyses for species determination and morphological classification to ascertain the developmental stage. The presence of *A. phagocytophilum* was determined using real-time PCR. Of the 1115 ticks analyzed from 4601 birds, 0.9% (*n* = 10), including *I. ricinus* and *Ixodes frontalis*, tested positive for *A. phagocytophilum*. Notably, common blackbirds (*Turdus merula*) yielded the highest number of *A. phagocytophilum*-infected ticks. The findings suggest that *A. phagocytophilum* is present in a small proportion of ticks infesting migratory birds in southeastern Sweden. Consequently, the role of birds as hosts for ticks infected with *A. phagocytophilum* appears to be low, suggesting that birds seem to play a minor indirect role in the geographic dispersal of *A. phagocytophilum*.

## 1. Introduction

Birds are commonly infested with various tick species [1], and both birds and ticks may harbor potentially human-pathogenic bacteria, viruses, and protozoa [2,3]. Since many birds are migratory, there is a potential risk for spread of pathogens to new areas along their migratory routes through ticks feeding on them [1,3]. The prevalence of tick infestation varies considerably between different species of birds, where bird feeding behavior, geographical location, climate, and season are among the factors introducing variation [3]. The highest prevalence of tick infestation is generally observed in ground-foraging bird species, which is closely linked to tick exposure levels [4]. Even though ticks can be transported by migratory birds, the establishment of new tick foci in an area depends on climate, vegetation, and the presence of suitable blood meal hosts, such as rodents [3]. Previous studies of ticks on birds show that *Ixodes ricinus* is the most common tick species infesting birds in Europe [5] and that most ticks collected from birds belong to immature life stages, i.e., larvae and nymphs [2,5,6]. However, it is important to note that several other tick species also feed on birds. Among them, ticks like *Ixodes frontalis* and *Ixodes arboricola* are ornithophagous [7].

*I. ricinus* has a wide distribution in Europe and serves as a vector for several human pathogens, including *Borrelia* spp. [8,9], *Anaplasma phagocytophilum* [9,10,11], *Babesia* spp. [12], *Rickettsia* spp. [2,9], *Neoehrlichia mikurensis* [13], and tick-borne encephalitis virus (TBEV) [9]. Over the years, numerous European studies have reported various tick-borne pathogens in ticks collected from birds, including *Borrelia burgdorferi* sensu lato group spirochetes, *A. phagocytophilum*, *Babesia divergens*, *Babesia venatorum*, *Coxiella burnetii*, and *N. mikurensis*, along with several *Rickettsia* spp., notably *Rickettsia helvetica*, and TBEV [8,12,14,15].

*Anaplasma phagocytophilum* is an intracellular bacterium that has been detected in mammals, birds, and ticks [5]. The bacterium uses mainly ticks in the *I. ricinus* complex as vectors, i.e., *I. ricinus* in Western Eurasia, *Ixodes persulcatus* in Eastern Eurasia, and *Ixodes scapularis* and *Ixodes pacificus* in North America [16]. Domestic ruminants are commonly infected with *A. phagocytophilum*. In these hosts, the disease is called tick-borne fever (TBF) and is characterized by symptoms such as high fever, dullness, abortion, and a drop in milk production, which results in economic losses for livestock owners [17]. *Anaplasma phagocytophilum* has also been detected in cats [18], dogs [19], birds [20], horses [21], and humans [22]. In humans, the disease is called human granulocytic anaplasmosis (HGA), with symptoms ranging from nonspecific influenza-like symptoms such as fever, headache, myalgia, and malaise to severe illness [17]. Cases of HGA have been described in Europe, North America, Asia, and Africa [16,17]. The prevalence of *A. phagocytophilum* in field-collected *I. ricinus* varies among countries and locations with a prevalence of 12% in Sweden [23], 24% in Denmark [24], 14% in Poland [25], and 6% in Norway [26]. The prevalence of *A. phagocytophilum* in *I. ricinus* ticks infesting birds is usually lower, below 5% [9,14,27,28,29]. Interestingly, the bacterium has also been detected in blood samples from passerine birds, suggesting that at least some birds might be capable of transmitting *A. phagocytophilum* to ticks [30]. However, the importance of birds for the transmission cycle of *A. phagocytophilum* is currently unclear.

Investigating the prevalence of a particular tick-borne pathogen is essential, not only for addressing public health concerns and veterinary implications but also for advancing our understanding of ecological dynamics, recognizing seasonal variations, and providing geographically specific information about the microorganism within ticks. This study specifically aims to evaluate the prevalence of *A. phagocytophilum* in ticks infesting birds during their spring and autumn migrations in the southeastern region of Sweden. 

## 2. Materials and Methods

### 2.1. Sampling, Analyses, and Processing of Ticks

For comprehensive details regarding the sampling site, bird capture procedures, bird classification, tick collection, and species determination, as well as nucleic acid extraction and cDNA synthesis, please refer to our previous report [8].

In summary, ticks were collected from birds captured between 15 March and 15 June, and between 15 July and 15 November 2009, at the Ottenby Bird Observatory, located on the southern tip of Öland Island in southeastern Sweden (56°12′ N, 16°24′ E). Each captured bird was identified to the species level. Subsequently, any ticks collected were photographed and morphologically classified according to their developmental stage (larva, nymph, or adult), and the sex of adults was determined. Individual ticks were homogenized using a TissueLyser II (Qiagen, Hilden, Germany), followed by extraction, purification, and isolation of total nucleic acids using the MagAttract^®^ Viral RNA M48 kit in a BioRobot M48 workstation (Qiagen, Hilden, Germany). The extracted nucleic acids were then reverse-transcribed to cDNA using the illustra™ Ready-to-Go RT-PCR Beads kit (GE Healthcare, Amersham Place, UK), serving as the template for all PCR assays. To determine the genus and species of the ticks, each specimen underwent PCR analysis targeting the tick mitochondrial *16S* rRNA gene, followed by DNA sequencing. 

The sampling of birds was approved by the Swedish Board of Agriculture, delegated through the Animal Research Ethics Committee in Linköping (decision 43–09).

### 2.2. Molecular Analyses of Anaplasma phagocytophilum in Ticks

Detection of *A. phagocytophilum* was carried out using a TaqMan real-time PCR assay, as previously described [31]. The primers ApF (5′–TTT TGG GCG CTG AAT ACG AT–3′) and ApR (5′–TCT CGA GGG AAT GAT CTA ATA ACG T–3′) and the probe ApM (FAM-TGC CTG AAC AAG TTATG-BHQ1) are designed to target the *A. phagocytophilum* citrate synthase gene (*gltA*) to amplify a 64-bp long amplicon. A 25 μL reaction mixture comprised 12.5 μL Maxima^®^ Probe qPCR Master Mix (2×) (Thermo Fisher Scientific, Waltham, MA, USA), 1.5 μL of each primer and0.375 μL of probe (10 μM; Invitrogen, Waltham, MA, USA), 7.125 μL RNase-free water, and 2 μL cDNA template. PCR reactions were conducted using a C1000™ Thermal Cycler and CFX96™ Real-Time PCR Detection System from Bio-Rad Laboratories, Inc. (Hercules, CA, USA), with an initial activation step at 95 °C for 10 min, followed by 40 cycles of 95 °C for 15 s and 60 °C for 1 min. For quality control, a synthetic plasmid containing the target sequence of the TaqMan real-time PCR assay was employed as a positive control. This plasmid contained the target sequence, spanning nucleotides 304–420 of the *A. phagocytophilum gltA* gene (GenBank: AF304137), synthesized and cloned into the pUC57 vector (GenScript, Piscataway, NJ, USA).

Subsequent confirmation analysis was performed on samples positive in the TaqMan qPCR assay by conventional PCR, using the first primer pair of the *A. phagocytophilum* specific *16S* PCR by Stuen et al. [32], producing an amplicon of 282 bp. Nucleotide sequencing of the PCR products was performed by Macrogen Inc. (Amsterdam, The Netherlands). All sequences obtained underwent confirmation by sequencing both strands. The resulting chromatograms were initially edited and analyzed using BioEdit Software v7.0 (Tom Hall, Ibis Therapeutics, Carlsbad, CA, USA), and the sequences were further scrutinized utilizing the Basic Local Alignment Tool (BLAST).

## 3. Results

### 3.1. Ticks Collected from Birds

A total of 4601 bird individuals of 65 species were examined for ticks at least once during the study period at the Ottenby Bird Observatory. Among them, 749 bird individuals (comprising 759 bird captures) belonging to 35 species were found to be infested with a total of 1339 ticks. These findings were previously documented by Wilhelmsson et al. 2020, with detailed data available in Table 1 of the referenced publication [8].

In this study, a total of 1115 ticks (cDNA samples) were available for analysis. Among these, 533 were larvae, 561 were nymphs, two were adult females, and 19 ticks could not be classified into a developmental stage due to technical constraints. The ticks subjected to analysis (Table 1) were identified through molecular methods, with 1042 identified as *I. ricinus* (consisting of 501 larvae, 534 nymphs, and seven specimens of unknown developmental stage), 23 as *I. frontalis* (comprising eight larvae, 14 nymphs, and one adult female), and 22 ticks designated solely as *Ixodes* spp. (including 12 larvae and ten nymphs). Additionally, 12 ticks were identified as *Haemaphysalis punctata* (eleven larvae, one nymph), and four ticks were recognized as *Hyalomma marginatum* (one larva, two nymphs, and one adult female). The remaining 12 ticks could not be molecularly identified to species or developmental stage due to inadequate sequences (despite multiple sequencing attempts) or the absence of accompanying photos.

The ticks analyzed in this study were removed from a total of 34 bird species (Table 2).

### 3.2. Prevalence of Anaplasma phagocytophilum in the Ticks

Of the 1115 ticks analyzed, ten (0.9%) were detected positive for *A. phagocytophilum* by real-time PCR (Table 1 and Table 2). Importantly, all infected ticks were removed from different individual birds. Six of the positive ticks were collected from common blackbirds *(Turdus merula*), two from European robins (*Erithacus rubecula*), one from a willow warbler (*Phylloscopus trochilus*), and one from a tree pipit (*Anthus trivialis*) (Table 2 and Table 3).

Seven of the *A. phagocytophilum*-positive ticks were *I. ricinus* (one larva and six nymphs), two were *I. frontalis* (one larva and one nymph), and one tick could neither be identified to species nor developmental stage (Table 3).

The ticks positive for *A. phagocytophilum* were mainly collected in April (*n* = 5) and most of them were half to fully fed (*n* = 7) (Table 3). According to the results of previous studies investigating the presence of *Borrelia* bacteria and *Babesia* protozoa in the same tick material as in the present study [8,12], three of the *A. phagocytophilum*-positive ticks were co-infected with *Borrelia valaisiana* (*n* = 1), *Borrelia turdi* (*n* = 1), and *Borrelia miyamotoi* (*n* = 1) (Table 3), but none was co-infected with *Babesia* spp. 

We obtained DNA sequences from six out of the ten positive *A. phagocytophilum* samples. Four of these sequences were identical to each other and showed 100% sequence (257/257) identity with those of *A. phagocytophilum* (accession no. ON614171.1) detected in a tick collected in Serbia; one sequence showed 100% (260/260) sequence identity with *A. phagocytophilum* (accession no. MN252874.1) detected in a tick removed from a bird in Antikythira, Greece; and one sequence showed 99% (256/257) sequence identity with *A. phagocytophilum* (accession no. MT020436.1) that was found in various organs, including the heart, liver, spleen, lung, bone marrow, and blood-fed fleas and ticks collected from a Himalayan marmot (*Marmota himalayana* in China). Attempts to amplify the *16S* rRNA from the remaining four real-time PCR-positive samples were unsuccessful.

## 4. Discussion

Our study revealed a 0.9% prevalence of *A. phagocytophilum* in ticks collected from migratory birds on the island of Öland in southeastern Sweden. Most infected ticks were nymphs of *I. ricinus*, predominantly found on common blackbirds, with the highest incidence occurring during the spring. Notably, three of the *A. phagocytophilum*-infected ticks were also co-infected with *Borrelia* bacteria.

Consistent with other European studies on ticks in birds, nearly all the ticks collected in our study were identified as *I. ricinus* [9,11,20,28]. Moreover, all the *I. ricinus* ticks we encountered were subadults, aligning with previous research that highlights a preference among nymphs and larvae of *I. ricinus* for birds, particularly in ground-foraging species, as opposed to adult ticks that typically favor larger mammals and rarely feed on birds or small mammals [9,14,20,33,34]. Only two adult female ticks were found, one *I. frontalis* and one *H. marginatum.*

Other tick species, like *H. punctata* and *H. marginatum* may also infest birds. However, these species were less common, and in total, less than 20 non-*Ixodes* ticks were found in our study. Adult *H. punctata* prefer medium to large mammals such as domestic ungulates and are rarely found on birds, while immatures are more commonly found on small mammals and birds [6,35,36]. This was also the case in our study where most of the *H. punctata* were larvae. Every year, birds infested with *H. marginatum* are transported from Southern Europe to Sweden and other countries located on the same latitude [7]. Birds and small mammals are known to host *H. marginatum* larvae and nymphs, while adult ticks prefer both wild and domestic ungulates [37]. In our study, at least one tick of each developmental stage of *H. marginatum* was recorded, indicating that all stages can be transported to Sweden by birds. Previous studies have shown that *H. punctata* constitutes a minor risk in pathogen transmission from ticks to humans since this tick species rarely bite humans [7,36]. Our results confirm other studies showing that *H. marginatum* ticks are not infected by *A. phagocytophilum*. This suggests that the interaction between *H. marginatum* ticks and migratory birds plays an insignificant role in the ecology of *A. phagocytophilum* [10,38].

*Anaplasma phagocytophilum* appears to be present in *I. ricinus* in all countries across Europe [17], with a prevalence range varying widely from 0.9% to 8.0% between different countries and studies [9,14,20,23,28]. The prevalence among ticks collected from migratory birds in Southern Norway was 0.9% [14], consistent with our study’s findings. In the present study, all ticks testing positive for *A. phagocytophilum* were removed from ground-foraging birds, aligning with findings from previous studies highlighting the significance of ground-foraging birds, particularly thrushes, as key hosts for *A. phagocytophilum*-infected ticks [9,11,28]. However, these findings could be attributed to the tendency of ground-foraging bird species to contract a greater number of ticks, rather than indicating a preference of *A. phagocytophilum* for specific bird species. The presence of the bacterium has also been detected in blood samples from passerine birds, including Blackbird, Chaffinch, House Sparrow, Spanish Sparrow, Rock Bunting, Woodchat Shrike, Magpie, and Long-tailed Tit [30]. This suggests the possibility of certain avian species serving as hosts for transmitting *A. phagocytophilum* to ticks. Previous studies on the prevalence of *A. phagocytophilum* in ticks collected from migrating birds have demonstrated cases where larvae were infected with *A. phagocytophilum* [20,34], supporting the findings of our study where two *Ixodes* larvae were infected—one *I. ricinus* and one *I. frontalis*. Since transovarial transmission of *A. phagocytophilum* has not been observed [17,34], this implies that certain birds, particularly in terms of transmission to *I. ricinus*, might function as competent reservoirs or transmission hosts. Targeted investigations into avian blood could serve to delve deeper into this hypothesis. Nevertheless, the positive larvae in our study, as well as in other studies, could potentially have acquired their infections through tick co-feeding.

Three of the *A. phagocytophilum* infected ticks were co-infected by *Borrelia* species (*B. valaisiana*, *B. miyamotoi*, and *Borrelia turdi,* respectively). This is in line with previous studies on infected *I. ricinus* ticks collected from migrating birds in Switzerland and Norway presenting co-infection between *B. garinii* and *A. phagocytophilum* [9,14]. Despite the large sample volumes, both in this study and in previous ones, the number of co-infected ticks remained low [9,14]. However, co-infections must always be considered, especially in regions known for high prevalence of various tick-borne pathogens [39]. Humans may be co-infected with tick-borne pathogens in two ways; either because of a tick bite from a multi-infected tick containing more than one pathogen, or because of more than one tick bite from ticks containing different pathogens [39].

While the presence of *A. phagocytophilum* in a tick is a concern due to its potential to transmit the bacterium to other hosts, it does not necessarily imply an immediate health risk for humans or other animals. The ability of *A. phagocytophilum* to infect different hosts and cause distinct diseases means that not all strains are equally virulent to humans or other species. The fact that *A. phagocytophilum* contains biologically distinct strains [40] that infect different host species, leading to human granulocytic anaplasmosis, equine granulocytic anaplasmosis, canine granulocytic anaplasmosis, and tick-borne fever, underscores the complexity of the interactions between the bacterium, its hosts, and the vectors involved. This diversity in strains and host specificity indicates that *A. phagocytophilum* has adapted to various mammalian hosts, and its pathogenicity may vary depending on the specific strain and host involved. Unfortunately, we did not conduct an analysis to characterize the strain of *A. phagocytophilum* detected in the present paper.

Another limitation of the study is the small number of *Anaplasma*-positive samples, coupled with the fact that the sampling took place 15 years ago, a constraint we acknowledge. Additionally, four out of ten *A. phagocytophilum*-positive specimens failed to amplify the *16S* rRNA gene using conventional PCR assays. It is possible that these ticks contained a lower amount of *A. phagocytopilum* bacteria compared to ticks containing typeable *A. phagocytophilum* which may, at least partly, explain why PCR products, used to determine *A. phagocytophilum*, were not amplified in the conventional PCR assays.

## 5. Conclusions

This study reveals that the bacterium *A*. *phagocytophilum* is present in ticks infesting migratory birds both in spring and in autumn capture, in southeastern Sweden, which could lead to dissemination of these tick-borne microorganisms into new areas. However, the importance of birds as transmission hosts of *A*. *phagocytophilum* seems to be minor, although this is still unclear and needs further investigation.

## Figures and Tables

**Table 1:** Presence of *Anaplasma phagocytophilum* in different tick species and life stages.

	No. of Ticks (No. of PCR-Positive for *Anaplasma phagocytphilum*)						
Tick Life Stage	*Haemaphysalis punctata*	*Hyalomma marginatum*	*Ixodes ricinus*	*Ixodes frontalis*	*Ixodes* spp.	Unknown ^1^	Total
Larva	11 (0)	1 (0)	501 (1)	8 (1)	12 (0)	0 (0)	533 (2)
Nymph	1 (0)	2 (0)	534 (6)	14 (1)	10 (0)	0 (0)	561 (7)
Adult female	0 (0)	1 (0)	0 (0)	1 (0)	0 (0)	0 (0)	2 (0)
Unknown	0 (0)	0 (0)	9 (0)	0 (0)	0 (0)	12 (1)	19 (1)
Total	12 (0)	4 (0)	1042 (7)	23 (2)	22 (0)	12 (1)	1115 (10)

^1^ The tick species and life stage remain undetermined, as the photograph of the tick is missing. Additionally, molecular identification was not carried out due to technical issues.

**Table 2:** Presence of *Anaplasma phagocytophilum* in ticks removed from different bird species.

Bird Species	No. of Ticks (No. of AP-Positive Ticks) ^1^
*Accipiter nisus*	1 (0)
*Acrocephalus palustris*	4 (0)
*Acrocephalus scirpaceus*	1 (0)
*Anthus trivialis*	84 (1)
*Carduelis cannabina*	1 (0)
*Carduelis flammea*	5 (0)
*Certhia familiaris*	4 (0)
*Cyanistes caeruleus*	7 (0)
*Emberiza schoeniclus*	1 (0)
*Erithacus rubecula*	495 (2)
*Fringilla coelebs*	8 (0)
*Fringilla montifringilla*	1 (0)
*Hippolais icterina*	2 (0)
*Lanius collurio*	3 (0)
*Luscinia luscinia*	27 (0)
*Luscinia svecica*	1 (0)
*Motacilla alba*	3 (0)
*Oenanthe oenanthe*	1 (0)
*Parus major*	2 (0)
*Passer domesticus*	1 (0)
*Phoenicurus ochruros*	2 (0)
*Phoenicurus phoenicurus*	60 (0)
*Phylloscopus collybita*	2 (0)
*Phylloscopus trochilus*	54 (1)
*Prunella modularis*	16 (0)
*Regulus regulus*	8 (0)
*Sturnus vulgaris*	3 (0)
*Sylvia atricapilla*	4 (0)
*Sylvia communis*	43 (0)
*Sylvia curruca*	10 (0)
*Troglodytes troglodytes*	95 (0)
*Turdus iliacus*	20 (0)
*Turdus merula*	126 (6)
*Turdus philomelos*	20 (0)
**Total**	**1115 (10)**

^1^ AP-positive = *Anaplasma phagocytophilum*-positive tick detected by real-time PCR.

**Table 3:** Characteristics of *Anaplasma phagocytophilum*-positive ticks removed from birds.

Bird Species	Month of Collection	Tick Species	Tick Life Stage	Degree of Blood Engorgement	Co-Infection ^2^
Blackbird (*Turdus merula*)	March	NA ^1^	NA ^1^	NA ^1^	*Borrelia valaisiana*
Blackbird (*Turdus merula*)	March	*I. frontalis*	L	HF	
Blackbird (*Turdus merula*)	April	*I. ricinus*	N	LF	
Blackbird (*Turdus merula*)	April	*I. ricinus*	N	FF	
Blackbird (*Turdus merula*)	April	*I. ricinus*	N	FF	*Borrelia turdi*
European robin (*Erithacus rubecula*)	April	*I. ricinus*	L	FF	
Tree pipit (*Anthus trivialis*)	April	*I. ricinus*	N	LF	*Borrelia miyamotoi*
Willow warbler (*Phylloscopus trochilus*)	August	*I. ricinus*	N	HF	
European robin (*Erithacus rubecula*)	September	*I. ricinus*	N	FF	
Blackbird (*Turdus merula*)	November	*I. frontalis*	N	HF	

^1^ NA, not available. The tick species, life stage, and the degree of blood engorgement remain undetermined, as the photograph of the tick is missing. Additionally, molecular identification was not carried out due to technical issues. ^2^ The samples underwent prior analysis using real-time PCR to detect *Borrelia burgdorferi* sensu lato, as described in [8]. Abbreviations: L, larva; N, nymph; LF, little fed; HF, half fed; FF, fully fed.

## Data Availability

The data supporting the conclusions of this article are included within the article. Raw data can be shared with researchers upon a specific request.
